# A Board of Examiners in Midwifery

**Published:** 1900-06

**Authors:** 


### A BOARD OF EXAMINERS IN MIDWIFERY.

We quite agree with the subjoined editorial from the *Medical Record* of April 21st :

A bill has passed both Houses of the Legislature of this State, authorising the creation of a Board of Examiners in Midwifery in this city, composed of the assistant sanitary superintendents of the Board of Health. As representing the medical profession of the State, we have protested against the enactment of this measure, for the reasons, first, that the practice of midwifery, being a part of the practice of medicine, should be restricted to registered practitioners of medicine; the legalising of this practise by others will tend to perpetuate a system which we hope in time to succeed in having abolished; and second, if by any train of circumstances it should be considered advisable to legalise this practise by other than registered physicians, we believe any act of legislature with this object in view should apply to the whole state, and not be restricted to one city or county. We believe, if midwives are to be examined and licensed, the work of examining them should be placed in the hands of a State Board of Medical Examiners.

Happily this question will settle itself in favor of the medical profession, regardless of legal enactments, through supplantation of midwives by the physicians connected with obstetrical institutions.—*Obstetrics.*

---

Admission to the Paris Exposition for Members of the Medical Congress.—Dr. H. B. Jacobs, secretary of the American National Committee of the Thirteenth International Medical Congress, informs us that during the week of the International Medical Congress, August 2d to 9th, free admission to the Paris Exposition will be granted to members of the Congress. To secure this, members will receive a special card by applying to the office of the Congress, 21 Rue de l'École de Médecine.—*Boston Med. and Surg. Jour.*

---

The Section on Materia Medica of the American Medical Association.—The troubles of the American Medical Association are perennial. For years the annual quarrel surged around the permanent secretary, and now, no sooner has the permanent secretary been unseated and removed from the scene of conflict, than clouds gather round the section on materia medica. The American drug manufacturers are alarmed by a report that the section is to be captured by members of the American Medical Association who are on the side of German chemical houses, and who will turn the work of the section into an advertising bureau for foreign drugs. The medical press is urged to raise its voice to thwart this nefarious scheme. But surely this alarm must be without foundation in fact. The American firms seem to have forgotten that every member of the American Medical Association is an avowed upholder of the code of ethics, and no one whose life is regulated by this moral law could descend so far as to bamboozle his fellows in this unprincipled manner.—*Med. Record.*

---

Medical Association of the County of New York no Longer Independent.—At a special meeting held May 11th, the Medical Association of the County of New York voted away its independence by adopting a resolution dictated by the council of the State Medical Association, by which it is constituted a subordinate county association of the State Association, and binds itself to obey the laws, rules and regulations of the latter.—*Boston Med. and Surg. Jour.*

